# Enstrophy transport conditional on local flow topologies in different regimes of premixed turbulent combustion

**DOI:** 10.1038/s41598-017-11650-x

**Published:** 2017-09-14

**Authors:** Vassilios Papapostolou, Daniel H. Wacks, Nilanjan Chakraborty, Markus Klein, Hong G. Im

**Affiliations:** 1 University of Newcastle, School of Mechanical and Systems Engineering, Newcastle, NE1 7RU UK; 2 Durham University, School of Engineering and Computing Sciences, Durham, DH1 3LE UK; 3 Universität der Bundeswehr München, Fakultät für Luft- und Raumfahrttechnik, München, 85577 Neubiberg Germany; 4 King Abdullah University of Science and Technology (KAUST), Clean Combustion Research Center, Thuwal, 23955-6900 Saudi Arabia

## Abstract

Enstrophy is an intrinsic feature of turbulent flows, and its transport properties are essential for the understanding of premixed flame-turbulence interaction. The interrelation between the enstrophy transport and flow topologies, which can be assigned to eight categories based on the three invariants of the velocity-gradient tensor, has been analysed here. The enstrophy transport conditional on flow topologies in turbulent premixed flames has been analysed using a Direct Numerical Simulation database representing the corrugated flamelets (CF), thin reaction zones (TRZ) and broken reaction zones (BRZ) combustion regimes. The flame in the CF regime exhibits considerable flame-generated enstrophy, and the dilatation rate and baroclinic torque contributions to the enstrophy transport act as leading order sink and source terms, respectively. Consequently, flow topologies associated with positive dilatation rate values, contribute significantly to the enstrophy transport in the CF regime. By contrast, enstrophy decreases from the unburned to the burned gas side for the cases representing the TRZ and BRZ regimes, with diminishing influences of dilatation rate and baroclinic torque. The enstrophy transport in the TRZ and BRZ regimes is governed by the vortex-stretching and viscous dissipation contributions, similar to non-reacting flows, and topologies existing for all values of dilatation rate remain significant contributors.

## Introduction

Turbulent flows are inherently rotational in nature and the extent of this rotation is often quantified in terms of vorticity $\overrightarrow{\omega }$ (i.e. curl of velocity vector $\overrightarrow{u}$). An important and characteristic feature of turbulent flows is the vortex-stretching mechanism, which is responsible for spreading turbulent velocity fluctuations over different length scales^1^. Thus, fundamental understanding of the enstrophy (i.e. ${\rm{\Omega }}=\overrightarrow{\omega }\cdot \overrightarrow{\omega }\mathrm{/2}$) transport is of key importance for the purpose of characterisation of the energy cascade in turbulent flows. In order to better understand premixed flame-turbulence interaction, the seemingly chaotic turbulence may be split into different generic topologies. Understanding which topologies make significant contribution to the enstrophy transport in different regimes of combustion might therefore help to gain deeper insight into strongly coupled phenomena of chemical reaction and turbulent flow and mixing.The current analysis aims to identify the canonical flow configurations which make dominant contributions to the enstrophy transport in different regions of the flame brush for different combustion regimes. This information can be utilised to design simplified experimental configurations representing dominant flow topologies to gain further insight into the flame-turbulence interaction.

Turbulent flows exhibit eight generic canonical local flow configurations underneath an apparently random fluid motion^2, 3^, and these distinct flow topologies are categorised depending on the values of first, second and third invariants (i.e. *P*, *Q* and *R*, respectively) of the velocity gradient tensor, *A*_*ij*_ = ∂*u*_*i*_/∂*x*_*j*_ = *S*_*ij*_ + *W*_*ij*_, where the symmetric strain-rate tensor is *S*_*ij*_ = 0.5(*A*_*ij*_ + *A*_*ji*_) and the anti-symmetric rotation rate tensor is *W*_*ij*_ = 0.5(*A*_*ij*_ − *A*_*ji*_). Three eigenvalues of *A*_*ij*_, *λ*_1_, *λ*_2_, and *λ*_3_, are the solutions of the characteristics equation *λ* ^3^ + *Pλ* ^2^ + *Qλ *+ *R *= 0 with its invariants *P*, *Q* and *R* as specified below^2^:

1 $$P=-tr({A}_{ij})=-({\lambda }_{1}+{\lambda }_{2}+{\lambda }_{3})=-{S}_{ii}$$

2 $$Q=\mathrm{0.5(}{P}^{2}-{S}_{ij}{S}_{ij}+{W}_{ij}{W}_{ij})={Q}_{S}+{W}_{ij}{W}_{ij}/2$$

3 $$R=(-{P}^{3}+3PQ-{S}_{ij}{S}_{jk}{S}_{ki}-3{W}_{ij}{W}_{jk}{S}_{ki})/3$$

**Figure 1:**
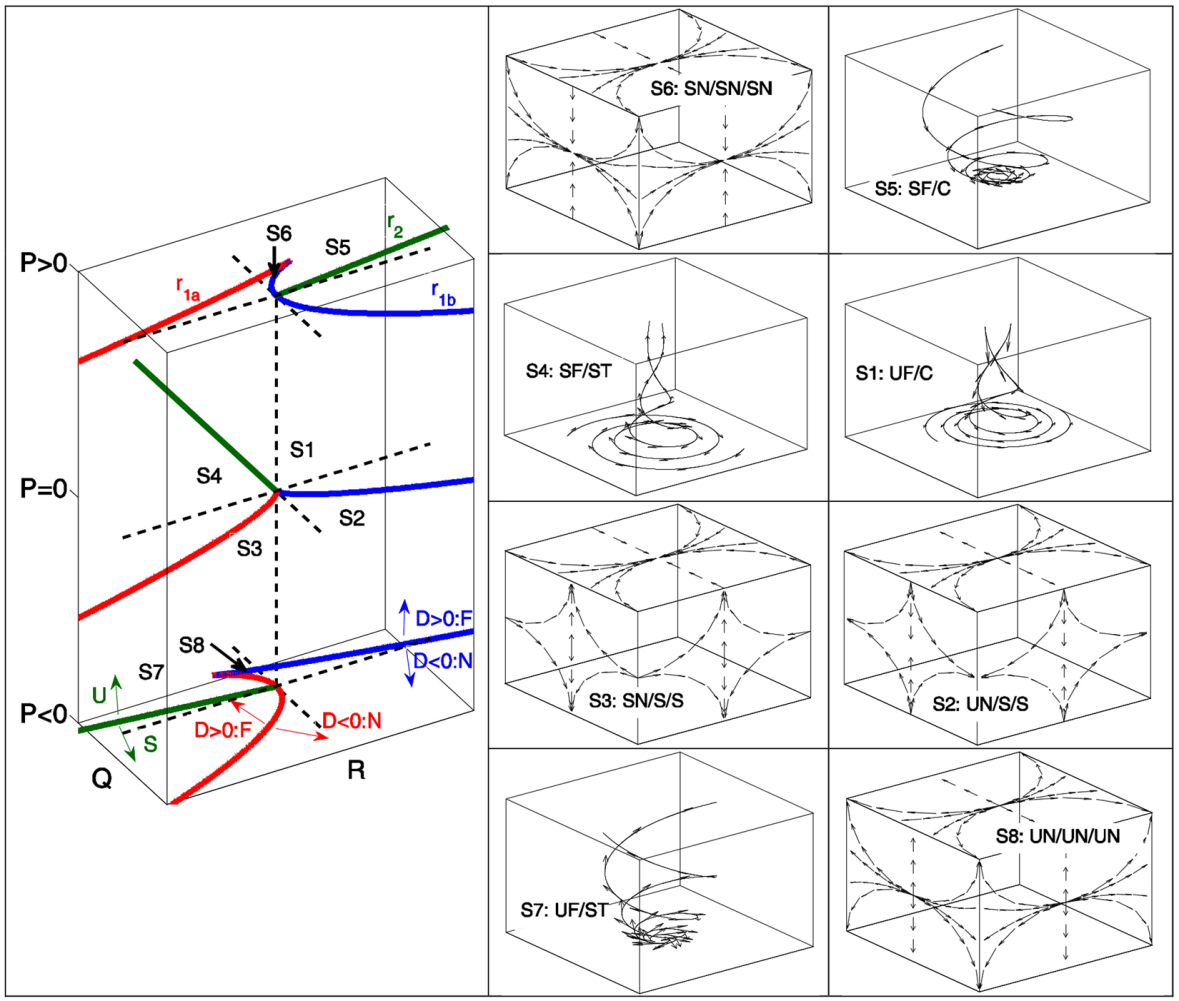
(Left) Classification of S1–S8 topologies in the *P* − *Q* − *R* space. The lines *r*_1*a*_ (red), *r*_1*b*_ (blue) and *r*_2_ (green) dividing the topologies are shown. Black dashed lines correspond to *Q* = 0 and *R* = 0 and *Q* = *R* = 0. (Right) Classification of S1–S8 topologies: UF = unstable focus, UN = unstable node, SF = stable focus, SN = stable node, S = saddle, C = compressing, ST = stretching. For *P* = 0 all topologies (S1, S2, S3, S4) are shown. For *P* > 0 (*P *< 0) only the additional topologies S5, S6 (S7, S8) are shown. The sign of the discriminant *D* (dividing the *Q* − *R* plane in focal and nodal topologies) as well as the (un)stable regions are indicated with arrows.

The discriminant, *D* = [27*R* ^2^ + (4*P* ^3^ − 18*PQ*)*R *+ 4*Q* ^3^ − *P* ^2^ *Q* ^2^]/108, of the characteristic equation *λ* ^3^ + *Pλ* ^2^ + *Qλ *+ *R *= 0 divides the *P* − *Q* − *R* phase-space into two regions: the focal (*D *> 0) and nodal (*D *< 0) topologies^2, 3^. The *A*_*ij*_ tensor exhibits one real eigenvalue and two complex conjugate eigenvalues for focal topologies, whereas *A*_*ij*_ shows three real eigenvalues for nodal topologies. The surface *D* = 0 leads to two subsets *r*_1*a*_ and *r*_1*b*_ in *P* − *Q* − *R* phase space which are given by Refs. 2 and 3: *r*_1*a*_ = *P*(*Q* − 2*P* ^2^/9)/3 − 2(−3*Q *+ *P* ^2^)^3/2^/27 and *r*_1*b*_ = *P*(*Q* − 2*P* ^2^/9)/3 + 2(−3*Q *+ *P* ^2^)^3/2^/27. In the region *D* > 0, *A*_*ij*_ has purely imaginary eigenvalues on the surface *r*_2_, which are given by *R* = *PQ*. The surfaces *r*_1*a*_, *r*_1*b*_ and *r*_2_, where *r*_2_ is desc*r*ibed by *PQ* − *R *= 0, divide the *P* − *Q* − *R* phase space into eight generic flow topologies, referred to as S1-S8, as shown schematically in Fig. 1 ^2, 3^. To date, a large body of literature^4–11^ concentrated on the local flow topology distribution from various viewpoints for incompressible fluids where $P=-\nabla \cdot \overrightarrow{u}=0$. However, relatively limited effort has been given to local flow topology distribution in compressible flows (i.e. *P *≠ 0)^12–15^. Only recently attention was given to turbulent reacting flows^16–21^ where *P* attains non-negligible magnitude even for small values of Mach number and predominantly *P *< 0 is obtained due to positive dilatation rate as a result of chemical heat release ($\nabla \cdot \overrightarrow{u} > 0$). Tanahashi *et al*.^16^ used the second invariant *Q* to distinguish between strain-dominated and vorticity-dominated regions in premixed turbulent combustion. Cifuentes and co-workers^18, 19^ demonstrated that the probability of finding focal topologies decreases from the unburned to the burned gas side of the flame front, which was also observed by Wacks *et al*.^20^ for droplet combustion. A recent analysis by Wacks *et al*.^21^ focused on local flow topology distributions for a direct numerical simulations (DNS) database consisting of three nominally thermo-diffusively neutral *H*_2_-air turbulent premixed flames representative of the corrugated flamelets, thin reaction zones and broken reaction zones regimes^22^. This analysis revealed significant qualitative differences in flow topology distribution within the flame front for flames representing different regimes of premixed turbulent combustion.

In turbulent flow, the enstrophy (i.e. Ω = *ω*_*i*_ *ω*_*i*_/2 where *ω*_*i*_ is the *i* ^*th*^ component of vorticity) and vorticity fields affect the statistical behaviours of the second and third invariants, *Q* and *R*, which in turn affect the distributions of local flow topologies S1–S8. The instantaneous transport equation of Ω = *ω*_*i*_ *ω*_*i*_/2 is given by^23–30^:

4 $$\frac{\partial {\rm{\Omega }}}{\partial t}+{u}_{k}\frac{\partial {\rm{\Omega }}}{\partial {x}_{k}}=\mathop{\underbrace{{\omega }_{i}{\omega }_{k}\frac{\partial {u}_{i}}{\partial {x}_{k}}}}\limits_{{T}_{I}}\mathop{\underbrace{-{\varepsilon }_{ijk}\frac{{\omega }_{i}}{{\rho }^{2}}\frac{\partial \rho }{\partial {x}_{j}}\frac{\partial {\tau }_{kl}}{\partial {x}_{l}}}}\limits_{{T}_{II}}\mathop{\underbrace{+{\varepsilon }_{ijk}\frac{{\omega }_{i}}{\rho }\frac{{\partial }^{2}{\tau }_{kl}}{\partial {x}_{j}\partial {x}_{l}}}}\limits_{{T}_{III}}\mathop{\underbrace{-2\frac{\partial {u}_{k}}{\partial {x}_{k}}{\rm{\Omega }}}}\limits_{{T}_{IV}}\mathop{\underbrace{+{\varepsilon }_{ijk}\frac{{\omega }_{i}}{{\rho }^{2}}\frac{\partial \rho }{\partial {x}_{j}}\frac{\partial p}{\partial {x}_{k}}}}\limits_{{T}_{V}}$$

The term *T*_*I*_ is the vortex-stretching contribution to the enstrophy transport, whereas term *T*_*II*_ arises due to misalignment of gradients of density and viscous stresses. The term *T*_*III*_ is responsible for molecular diffusion and dissipation of enstrophy due to viscous action, whereas the term *T*_*IV*_ signifies the dissipation of enstrophy due to dilatation rate. The term *T*_*V*_ is the baroclinic torque term which arises due to misalignment between pressure and density gradients.

A number of recent studies^23–29^ investigated the Reynolds-averaged enstrophy transport equation in turbulent premixed flames by Reynolds-averaging eq. 4:

5 $$\frac{\partial \overline{{\rm{\Omega }}}}{\partial t}+\overline{{u}_{k}\frac{\partial {\rm{\Omega }}}{\partial {x}_{k}}}=\mathop{\underbrace{\overline{{\omega }_{i}{\omega }_{k}\frac{\partial {u}_{i}}{\partial {x}_{k}}}}}\limits_{{\overline{T}}_{I}}\mathop{\underbrace{-\overline{{\varepsilon }_{ijk}\frac{{\omega }_{i}}{{\rho }^{2}}\frac{\partial \rho }{\partial {x}_{j}}\frac{\partial {\tau }_{kl}}{\partial {x}_{l}}}}}\limits_{{\overline{T}}_{II}}\mathop{\underbrace{+\overline{{\varepsilon }_{ijk}\frac{{\omega }_{i}}{\rho }\frac{{\partial }^{2}{\tau }_{kl}}{\partial {x}_{j}\partial {x}_{l}}}}}\limits_{{\overline{T}}_{III}}\mathop{\underbrace{-\overline{2\frac{\partial {u}_{k}}{\partial {x}_{k}}{\rm{\Omega }}}}}\limits_{{\overline{T}}_{IV}}\mathop{\underbrace{+\overline{{\varepsilon }_{ijk}\frac{{\omega }_{i}}{{\rho }^{2}}\frac{\partial \rho }{\partial {x}_{j}}\frac{\partial p}{\partial {x}_{k}}}}}\limits_{{\overline{T}}_{V}}$$

Here $\bar{q}$ indicates the Reynolds-averaged value of a general quantity *q*. In the current analysis all the Reynolds averaged quantities are evaluated by ensemble averaging the quantities in the statistically homogeneous directions (which are the directions normal to the direction of the mean flame propagation) when the flame attains a quasi-steady state. A similar approach was adopted in several previous analyses (e.g. refs 26 and 27 and references therein).

Hamlington *et al*.^23^ concentrated on different mechanisms of vorticity generation and the alignment of vorticity with local principal strain rates in the flames representing the thin reaction zones regime of premixed turbulent combustion. Chakraborty^25^ showed that the alignment of vorticity with local principal strain rates is significantly affected by the regime of combustion and the characteristic Lewis number (i.e. ratio of thermal diffusivity to mass diffusivity). Both studies^23, 25^ reported a predominant alignment of vorticity with the intermediate principal strain rate in turbulent premixed flames, which is similar to non-reacting turbulent flows^31^. However, the relative alignment of vorticity with the most extensive and most compressive principal strain rates is significantly affected by the strength of dilatation rate^25^, which is influenced by the regime of combustion and the characteristic Lewis number. Hamlington *et al*.^23^ showed that enstrophy decays significantly in the burned gas across the flame brush, whereas Treurniet *et al*.^24^ demonstrated an opposite trend for the flames with high density ratio between the unburned and burned gases. This behaviour has been explained by Lipatnikov *et al*.^26^ by analysing the terms of enstrophy and vorticity transport equation for weakly turbulent premixed flames representing the corrugated flamelets regime.

Recent analyses by Chakraborty *et al*.^27^ and Dopazo *et al*.^30^ demonstrated that the characteristic Lewis number significantly affects the vorticity generation within the flame, and a combination of augmented flame normal acceleration and high extent of flame wrinkling for small values of Lewis number may give rise to a significant amount of vorticity generation within the flame brush due to baroclinic torque. Bobbit and coworkers^28, 29^ demonstrated that the enstrophy transport in statistically planar flames propagating in homogeneous isotropic turbulence for large values of Karlovitz number is governed by the relative balance between the vortex-stretching and viscous dissipation similar to its non-reacting counterpart, and also revealed that the choice of chemical reaction mechanism does not affect the qualitative nature of the enstrophy transport. The enstrophy field in turbulent premixed flames using cinema- stereoscopic particle image velocimetry (PIV) measurements of rim-stabilised turbulent premixed flames has been investigated^32–34^ and confirmed some of the observations based on DNS data.

The aforementioned analyses^23–29, 31–34^ provided invaluable insight into the flame-turbulence interaction, but the nature of the enstrophy transport conditional on generic local flow topologies S1–S8 in different premixed combustion regimes is yet to be analysed in detail. Such an analysis is expected to reveal the canonical flow configurations which make dominant contributions to the enstrophy transport for different combustion regimes.

In order to address the aforementioned gap in the existing literature and to meet the above objectives an existing detailed chemistry DNS database^21, 35^ has been considered consisting of three *H*_2_-air flames with an equivalence ratio of *ϕ* = 0.7, representative of the combustion processes in the corrugated flamelets, thin reaction zones, and broken reaction zones regimes of combustion. A detailed chemical mechanism^36^ involving 9 steps and 19 chemical reactions is considered for this analysis. The unburned gas temperature *T*_0_ is taken to be 300 K, which gives rise to an unstrained laminar burning velocity *S*_*L*_ = 135.62 cm/s under atmospheric pressure. The inlet values of normalised root-mean-square turbulent velocity fluctuation *u*′/*S*_*L*_, turbulent length scale to flame thickness ratio *l*_*T*_/*δ*_*th*_, Damköhler number *Da *= *l*_*T*_ *S*_*L*_/*u*′*δ*_*th*_, Karlovitz number *Ka *= (*ρ*_0_ *S*_*L*_ *δ*_*th*_/*μ*_0_)^0.5^(*u*′/*S*_*L*_)^1.5^(*l*_*T*_/*δ*_*th*_)^−0.5^ and turbulent Reynolds number *Re*_*t*_ = *ρ*_0_ *u*′*l*_*T*_/*μ*_0_ for all cases are presented in Table 1, where *ρ*_0_ is the unburned gas density, *μ*_0_ is the unburned gas viscosity, *l*_*T*_ is the most energetic length scale, *δ*_*th*_ = (*T*_*ad*_ − *T*_0_)/*max*|∇*T*|_*L*_ is the thermal flame thickness with *T*, *T*_*ad*_ and *T*_0_ being the instantaneous dimensional temperature, adiabatic flame temperature and unburned gas temperature, respectively, and the subscript ‘*L*’ is used to refer to unstrained laminar flame quantities.

**Table 1:** List of inflow turbulence parameters.

Case	A	B	C
*u*′/*S*_*L*_	0.7	5	14
*l*_*T*_/*δ*_*th*_	14	14	4
*Da*	20	2.8	0.29
*Ka*	0.75	14.4	126
*Re*_*t*_	227	1623	1298

The cases investigated in this study are representative of three regimes of combustion: case A: corrugated flamelets (*Ka* < 1), case B: thin reaction zones (1 < *Ka *< 100) and case C: broken reaction zones regime (*Ka *> 100)^22^. The Karlovitz number can be scaled as $Ka\sim {\delta }_{th}^{2}/{\eta }^{2}$ (where *η* is the Kolmogorov length scale), which suggests that the turbulent eddies do not disturb the inner flame structure in case A, whereas turbulent eddies penetrate into the preheat zone in the thin reaction zones regime, but the reaction zone (with typical thickness of *δ*_*th*_/10) retains a quasi-laminar structure. In the broken reaction zones regime, the energetic turbulent eddies may penetrate into the reaction zone and disturb the chemical processes.

## Results and Discussion

**Figure 2:**
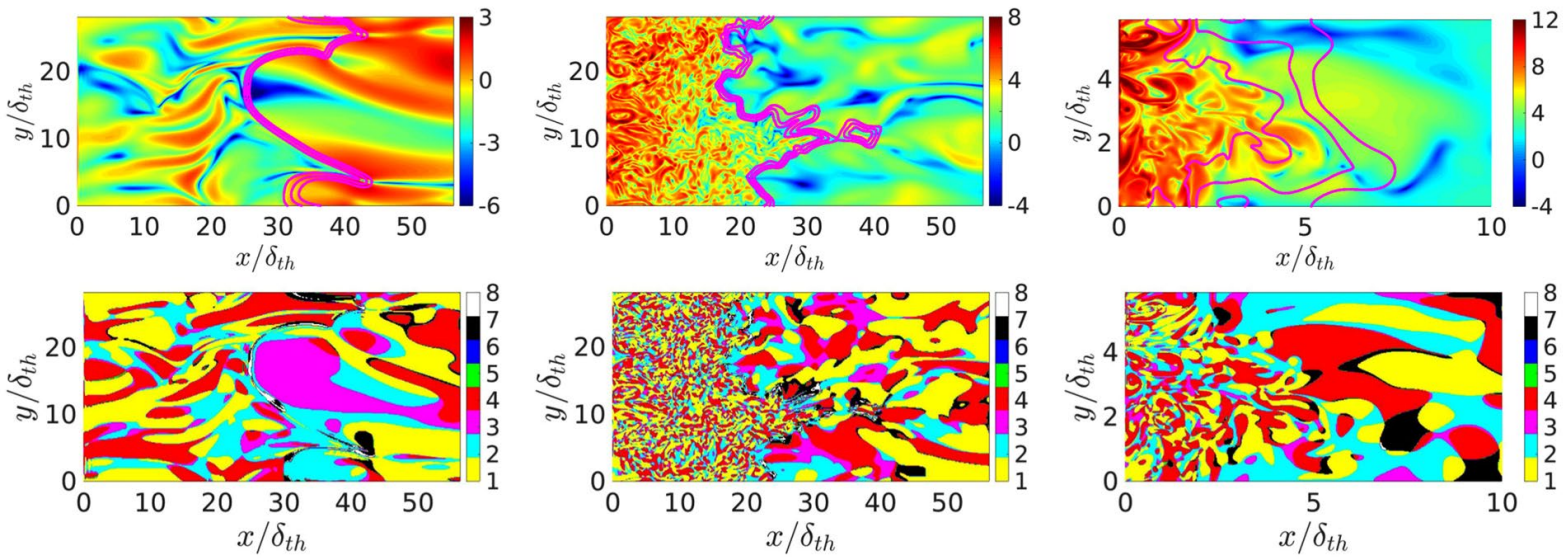
(Top row) Natural logarithm of instantaneous normalised enstrophy (Ω × (*δ*_*th*_/*S*_*L*_)^2^) fields: (left to right) case A, case B and case C (truncated domain). *c*_*T*_-isosurfaces are shown for *c*_*T*_ = 0.1, 0.5, 0.7. (Bottom row) Instantaneous local flow topology fields: (left to right) case A, case B and case C (truncated domain).

The distributions of natural logarithm (to cover the wide dynamic range) of normalised enstrophy field (${\rm{\Omega }}\times {\delta }_{th}^{2}/{S}_{L}^{2}$) in the central *x*_1_ − *x*_2_ plane for cases A, B and C are shown in Fig. 2 (top row) where the contours of temperature-based reaction progress variable *c*_*T*_ = (*T* − *T*_0_)/(*T*_*ad*_ − *T*_0_) = 0.1, 0.5 and 0.7 are also shown. By definition, *c*_*T*_ increases from 0 in the unburned gases to 1.0 in fully burned products. The local distributions of S1-S8 topologies in the corresponding planes are shown in the bottom row of Fig. 2. It is clearly seen that there are significant qualitative differences in the enstrophy and topology distributions between these cases. For example, cases B and C show a much larger range of structures, whereas mostly large scale structures are evident in case A. As seen in Table 1, *Re*_*t*_ in case B is greater than in case A, whereas the value of *l*_*T*_ remains the same. Thus, case B shows smaller structures than case A. For case C, the length scale remains smaller than case A, which along with higher *Re*_*t*_ leads to smaller structures than in case A (note the different scaling of Case C).

**Figure 3:**
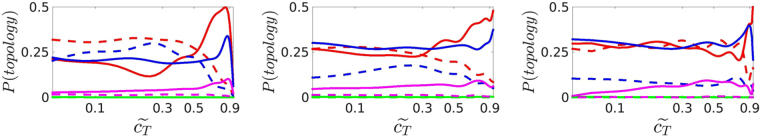
Variation of topology populations (by %) with ${\mathop{c}\limits^{ \sim }}_{T}$ ($0.05 < {\mathop{c}\limits^{ \sim }}_{T} < 0.95$) for cases (left to right) A-C: focal topologies S1 (red solid line), S4 (blue solid line), S5 (green solid line), and S7 (magenta solid line) and nodal topologies S2 (red dashed line), S3 (blue dashed line), S6 (green dashed line), and S8 (magenta dashed line). The *x*-axis is shown using a logarithmic scale.

Figure 2 (top row) further shows that in case A the enstrophy value increases locally from the unburned to the burned gas side of the flame due to flame-induced vorticity generation. In contrast, the magnitude of enstrophy decreases from unburned to burned gas side of the flame for cases B and C. Furthermore, Fig. 2 indicates that S1, S2, S3 and S4 topologies are predominantly present in the burned gas in all cases, but the topology distribution within the flame is significantly different between cases A–C. This can be substantiated from Fig. 3 which shows the probability of finding each flow topology for different values of Favre-averaged reaction progress variable ${\tilde{c}}_{T}$, where the Favre-average of a general quantity *q* is given by $\tilde{q}=\overline{\rho q}/\overline{\rho }$. In actual RANS simulations, one obtains Favre-averaged reaction progress variable ${\tilde{c}}_{T}$ and not the Reynolds averaged reaction progress variable ${\overline{c}}_{T}$. Furthermore, in statistically planar flames both Reynolds averaged and Favre-averaged values of reaction progress variable remain unique functions of the mean direction of flame propagation and thus all the terms in this configuration can be presented as a function of either ${\bar{c}}_{T}$ or ${\tilde{c}}_{T}$. The findings and conclusions would have been qualitatively similar if the results were shown as a function of ${\bar{c}}_{T}$. As ${\tilde{c}}_{T}$ is readily obtained from RANS simulations, the results are shown as a function of Favre-averaged reaction progress variable in this paper.

Figure 3 indicates that S1–S4 topologies remain major contributors within the flame front for all three cases. As shown in Fig. 1, S1–S4 appear for all values of *P*, and thus they remain dominant contributors, which is consistent with previous findings by Cifuentes *et al*.^19^. The dilatation rate $\nabla \cdot \overrightarrow{u}=-P$ is predominantly positive within the flame and thus the topologies S5 and S6, which are typical of positive *P* (i.e. $P=-\nabla \cdot \overrightarrow{u}$), rarely occur within the flame, and a similar observation was recently made by Cifuentes *et al*.^19^. The probability of obtaining S7 topology, which is typical of positive dilatation rate (i.e. $\nabla \cdot \overrightarrow{u}=-P > 0$), increases from the unburned to the burned gas side of the flame brush (i.e. with increasing ${\tilde{c}}_{T}$) in all cases considered here. The strength of dilatation rate and likelihood of obtaining high positive values of $\nabla \cdot \overrightarrow{u}$ are significantly smaller in case C than that in cases A and B because energetic turbulent eddies penetrate into the reaction zone and disturb the chemical reaction, which in turn reduces the heat release rate and the magnitude of $\nabla \cdot \overrightarrow{u}$ ^21^. The weakening of dilatation rate effects in case C is reflected in the fact that the S8 topology completely disappears in case C.

**Figure 4:**
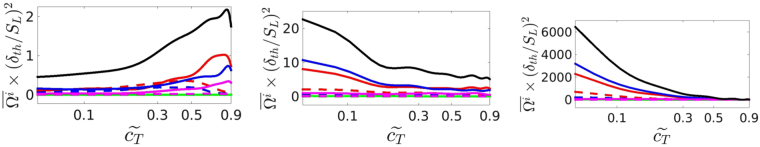
Variation of normalised $\overline{{{\rm{\Omega }}}^{i}}$ with ${\mathop{c}\limits^{ \sim }}_{T}$ ($0.05 < {\mathop{c}\limits^{ \sim }}_{T} < 0.9$), where {*i *= 0} are the total enstrophies (black solid lines) and {*i *= 1, ..., 8} are the percentage-topology-weighted enstrophies S1–8, respectively, for cases (left to right) A-C: focal topologies S1 (red solid line), S4 (blue solid line), S5 (green solid line), and S7 (magenta solid line) and nodal topologies S2 (red dashed line), S3 (blue dashed line), S6 (green dashed line), and S8 (magenta dashed line). The *x*-axis is shown using a logarithmic scale.

The variation of the normalised Reynolds-averaged enstrophy $\overline{{{\rm{\Omega }}}^{0}}\times {({\delta }_{th}/{S}_{L})}^{2}$ (where $\overline{{{\rm{\Omega }}}^{0}}=\overline{{\rm{\Omega }}}$ and $\overline{{{\rm{\Omega }}}^{i}}$ is defined such that $\overline{{{\rm{\Omega }}}^{{\rm{0}}}}={\sum }_{i\mathrm{=1}}^{8}\overline{{{\rm{\Omega }}}^{i}}$ where *i* = 1, 2, ..., 8 for S1–S8 respectively) conditional on ${\tilde{c}}_{T}$ is shown in Fig. 4. It is seen from Fig. 4 that $\overline{{{\rm{\Omega }}}^{0}}\times {({\delta }_{th}/{S}_{L})}^{2}$ increases for the major part of the flame brush from the unburned gas side towards the burned gas side, before decreasing towards the far edge of the burned gas side (${\tilde{c}}_{T}\approx 0.9$) in case A, whereas $\overline{{{\rm{\Omega }}}^{0}}\times {({\delta }_{th}/{S}_{L})}^{2}$ decreases across the flame brush for cases B and C, which is consistent with the observation made from Fig. 2. The decay of enstrophy from the unburned to the burned gas side of the flame brush for thermo-diffusively neutral flames within the thin reaction zones regime is consistent with previous findings^23, 27, 30^. Figure 4 also shows the contributions of each individual topology (i.e. $\overline{{{\rm{\Omega }}}^{i}}$ where *i *= 1, 2, ..., 8 for S1–S8) to the mean enstrophy as a function of ${\tilde{c}}_{T}$ for cases A-C, indicating that the topologies S1–S4 (with the focal topologies S1 and S4 as the leading contributors), which are obtained for all possible values of *P* (see Fig. 1), contribute significantly to the mean enstrophy in all cases. Furthermore, the focal topology S7, which occurs only for positive dilatation rates, contributes significantly to the mean enstrophy $\overline{{{\rm{\Omega }}}^{0}}$ in case A, but this trend weakens from case A to case C and the contribution of S7 remains negligible in case C.

**Figure 5:**
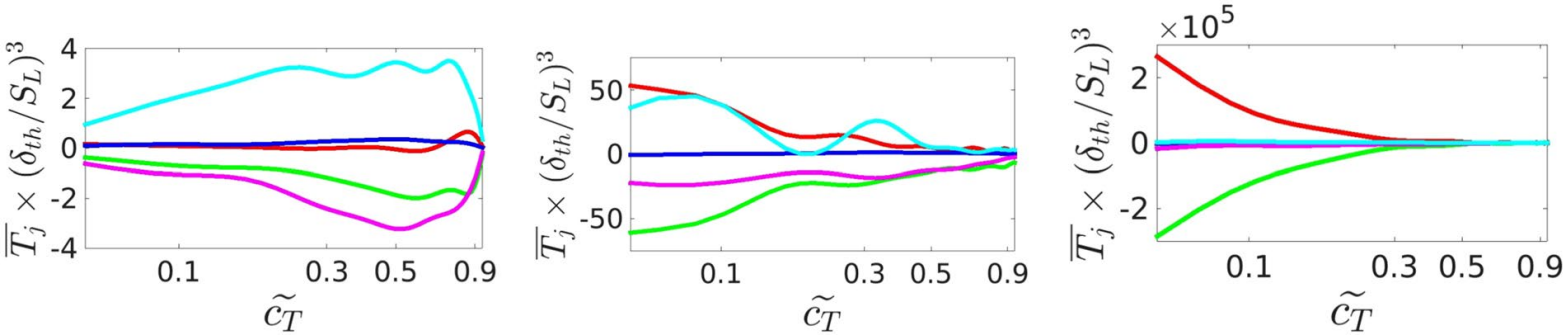
Variation with ${\mathop{c}\limits^{ \sim }}_{T}$ ($0.05 < {\mathop{c}\limits^{ \sim }}_{T} < 0.95$) of normalised enstrophy transport equation terms, $\overline{{T}_{j}}$ (*j* = *I*, *II*, *III*, *IV*, *V*), for (left to right) cases A-C: *T*_*I*_ (red line), *T*_*II*_ (blue line), *T*_*III*_ (green line), *T*_*IV*_ (magenta line), *T*_*V*_ (cyan line). The *x*-axis is shown using a logarithmic scale.

In order to identify the physical mechanisms responsible for the difference in the enstrophy statistics between cases A-C, which are representative of combustion situations in three different combustion regimes, the variation of ${\overline{T}}_{I}$, ${\overline{T}}_{II}$, ${\overline{T}}_{III}$, ${\overline{T}}_{IV}$ and ${\overline{T}}_{V}$ with ${\tilde{c}}_{T}$ for cases A-C are shown in Fig. 5. Significant differences are seen in the behaviour of the enstrophy transport between cases A, B and C. The term ${\overline{T}}_{III}$ due to the viscous diffusion and dissipation acts as a leading order sink, and the magnitude of the viscous torque contribution ${\overline{T}}_{II}$ remains small in comparison to ${\overline{T}}_{III}$ for all cases. In case A, the baroclinic torque contribution ${\overline{T}}_{V}$ acts as the major source term, and its magnitude remains comparable to that of ${\overline{T}}_{III}$. The contribution of the vortex-stretching term ${\overline{T}}_{I}$ remains weak in case A and its magnitude remains small in comparison to that for the terms of ${\overline{T}}_{III}$ and ${\overline{T}}_{V}$, whereas the dilatation term ${\overline{T}}_{IV}$ acts a major sink term in this case and its magnitude remains comparable to that of the combined viscous diffusion and dissipation term ${\overline{T}}_{III}$. The relative contributions of the dilatation term ${\overline{T}}_{IV}$ and the baroclinic torque term ${\overline{T}}_{V}$ are somewhat diminished in the flame representing the thin reaction zones regime (e.g. case B) and become negligible in the flame representing the broken reaction zones regimes of combustion (e.g. case C). In case B, the vortex-stretching term ${\overline{T}}_{I}$ and the combined viscous diffusion and dissipation term ${\overline{T}}_{III}$ remain the major source and sink terms, respectively. Although the baroclinic term ${\overline{T}}_{V}$ and the dilatation term ${\overline{T}}_{IV}$ continue to be source and sink terms in case B, their magnitudes are generally small in comparison to ${\overline{T}}_{I}$ and ${\overline{T}}_{III}$, respectively. In case C, the transport of enstrophy is determined by the source and sink contributions of the vortex-stretching term ${\overline{T}}_{I}$ and the combined viscous diffusion and dissipation term ${\overline{T}}_{III}$, respectively and the magnitudes of ${\overline{T}}_{II}$, ${\overline{T}}_{IV}$ and ${\overline{T}}_{V}$ remain negligible in comparison to ${\overline{T}}_{I}$ and ${\overline{T}}_{III}$. The behaviour of the enstrophy transport in case C is qualitatively similar to a non-reacting flow in this configuration. The effects of dilatation rate and density change on the background fluid motion weaken with increasing Karlovitz number *Ka*, and thus the influences of the dilatation and baroclinic terms (i.e. ${\overline{T}}_{IV}$ and ${\overline{T}}_{V}$) weaken from the corrugated flamelets regime to the thin reaction zones regime to the broken reaction zones regime. The weaker contributions of ${\overline{T}}_{IV}$ and ${\overline{T}}_{V}$ in the thin reaction zones and broken reaction zones regime flames are consistent with previous findings^27–30^.

The vortex-stretching term ${\overline{T}}_{I}$ can be expressed as: ${\overline{T}}_{I}=\overline{\mathrm{2(}{e}_{\alpha }co{s}^{2}\alpha +{e}_{\beta }co{s}^{2}\beta +{e}_{\gamma }co{s}^{2}\gamma ){\rm{\Omega }}}$ where *e*_*α*_, *e*_*β*_ and *e*_*γ*_ are the most extensive, intermediate and the most compressive principal strain rates respectively, and the angles between $\overrightarrow{\omega }$ and the eigenvectors associated with *e*_*α*_, *e*_*β*_ and *e*_*γ*_ are given by *α*, *β* and *γ*, respectively. It was shown by Chakraborty^25^ that $\overrightarrow{\omega }$ predominantly aligns with *e*_*β*_ (i.e. high probability of |*cosβ*| ≈ 1), but the extent of alignment with *e*_*α*_ increases in regions of high chemical heat release. The extent of alignment of $\overrightarrow{\omega }$ with *e*_*α*_ (*e*_*γ*_) decreases (increases) with decreasing Karlovitz number especially in corrugated flamelets regime flames^25^ and a similar qualitative behaviour has been observed here. Interested readers are referred to Ref. 25 for the relevant explanations. The probability of increased alignment of $\overrightarrow{\omega }$ with the most compressive (i.e. most negative) and intermediate principal strain rates is responsible for the weak contribution of ${\overline{T}}_{I}$ in case A. In premixed flames dilatation rate $\nabla \cdot \overrightarrow{u}$ remains predominantly positive as a result of thermal expansion due to heat release, which gives rise to a predominantly negative value of dilatation contribution ${\overline{T}}_{IV}=-\overline{\mathrm{2(}\nabla \cdot \overrightarrow{u}){\rm{\Omega }}}$. It was demonstrated by Wacks *et al*.^21^ that the magnitude and influence of dilatation rate weakens from case A to C and accordingly ${\overline{T}}_{IV}$ plays a progressively less important role from case A to case C. It has been shown earlier that the cosine of the angle between $\overrightarrow{\omega }$ and $\nabla p$ × $\nabla \rho /{\rho }^{2}$ remains mostly positive within premixed flames^26, 27^, and a similar behaviour has been found here (not shown for brevity), which gives rise to a positive contribution of the baroclinic torque in the mean sense (i.e. ${\overline{T}}_{V}$) for all cases. A comparison between the enstrophy transport behaviours in cases A–C reveals that the baroclinic torque is responsible for the enstrophy generation in case A, which gives rise to local augmentation of enstrophy across the flame in this case (see Fig. 2). In cases B and C, only the vortex-stretching term ${\overline{T}}_{I}$ acts as the major source and its magnitude decreases from the unburned to the burned gas side of the flame brush.

**Figure 6:**
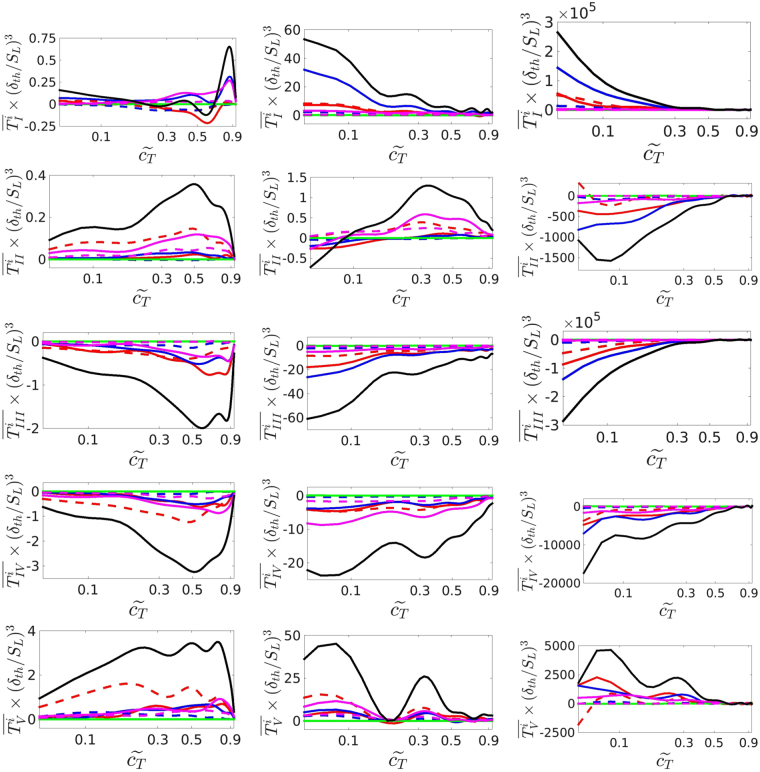
Variation of normalised $\overline{{T}_{j}^{i}}$ with ${\mathop{c}\limits^{ \sim }}_{T}$ ($0.05 < {\mathop{c}\limits^{ \sim }}_{T} < 0.95$), where {*i *= 0} are the total enstrophy transport equation terms (black solid lines) and {*i *= 1, ..., 8} are the percentage-topology-weighted terms corresponding to S1–8, respectively, for (top to bottom) terms {*j* = *I*, *II*, *III*, *IV*, *V*} and (left to right) cases A-C: focal topologies S1 (red solid line), S4 (blue solid line), S5 (green solid line), and S7 (magenta solid line) and nodal topologies S2 (red dashed line), S3 (blue dashed line), S6 (green dashed line), and S8 (magenta dashed line). The *x*-axis is shown using a logarithmic scale.

The term-by-term contributions of each individual topology to the enstrophy transport (i.e. ${\overline{T}}_{j}^{i}$ where *i *= 1, 2, …, 8 for S1–S8 and *j* = *I*, *II*, *III*, *IV*, *V* for ${\overline{T}}_{I}$, ${\overline{T}}_{II}$, ${\overline{T}}_{III}$, ${\overline{T}}_{IV}$ and ${\overline{T}}_{V}$, respectively, such that ${\overline{T}}_{j}={\overline{T}}_{j}^{0}={\sum }_{i\mathrm{=1}}^{8}{\overline{T}}_{j}^{i}$) as a function of ${\tilde{c}}_{T}$ for cases A-C are shown in Fig. 6. It is seen that the focal topology S7 (which is typical of *P *< 0) associated with stretching is a leading positive contributor to the vortex-stretching term ${\overline{T}}_{I}$ in the corrugated flamelets regime case A. Moreover, the focal topology S4 associated with stretching remains a significant positive contributor to the vortex-stretching term ${\overline{T}}_{I}$ in case A. In this case the magnitudes of the contributions of the topologies S1, S2 (partially obscured by S3) and S3 to the vortex-stretching term ${\overline{T}}_{I}$ remain significant, but these topologies act to induce negative values. The topologies S1, S2 and S4, which can be obtained irrespective of the value of *P*, play leading order roles in the vortex-stretching term ${\overline{T}}_{I}$ in the thin reaction zones regime case B and also in the broken reaction zones regime case C.

The topologies S2, S7, S8 and S4 contribute significantly to the viscous torque term ${\overline{T}}_{II}$ in the decreasing order of importance in the corrugated flamelets regime case A. The contribution of ${\overline{T}}_{II}$ remains positive in case A and mostly positive except towards the unburned gas side of the flame brush in the thin reaction zones regime case B. The topologies S1, S2, S8, S7 and S4 contribute significantly to ${\overline{T}}_{II}$ in case B and the positive contribution of ${\overline{T}}_{II}$ towards the burned gas side of the flame brush originates principally due to the S7 topology. The topologies S4, S1 and S2, which can be obtained irrespective of the value of *P*, contribute significantly to the viscous torque term ${\overline{T}}_{II}$ (in decreasing order of significance) in the broken reaction zones regime case C.

Figure 6 suggests that the S4, S1 and S2 topologies are the leading order contributors to the combined viscous diffusion and dissipation term ${\overline{T}}_{III}$ for all three cases, but S7 and, to a lesser extent, S3 topologies also contribute significantly to this term for the case representing the corrugated flamelets regime (i.e. case A). In the thin reaction zones and broken reaction zones regime cases (i.e. in cases B and C), S4, S1 and S2 topologies are responsible for most of the viscous diffusion and dissipation.

The S2, S7, S1, S4 and S8 (in decreasing order of significance) topologies contribute significantly to the dilatation rate ${\overline{T}}_{IV}$ and baroclinic torque ${\overline{T}}_{V}$ terms in the corrugated flamelets regime case A, whereas S7, S2, S1, S4 and S8 (in decreasing order of significance) topologies remain leading order contributors to ${\overline{T}}_{IV}$ in the thin reaction zones regime case B. The topologies S2, S7, S4, S1 and S8 (in decreasing order of significance) are the major contributors to the baroclinic torque ${\overline{T}}_{V}$ term in the thin reaction zones regime case B. In the broken reaction zones regime case C, the dilatation rate ${\overline{T}}_{IV}$ and the baroclinic torque ${\overline{T}}_{V}$ terms do not have much significance, but S4, S1 and S7 remain major contributors to these terms in this case.

The topologies which are significant contributors to ${\overline{T}}_{I}$, ${\overline{T}}_{II}$, ${\overline{T}}_{III}$, ${\overline{T}}_{IV}$ and ${\overline{T}}_{V}$ in cases A-C are summarised in Table 2. From the foregoing discussion and Table 2 it is evident that S1–S4, S7 and S8 topologies play key roles in the enstrophy transport in the corrugated flamelets regime flame, but the contributions of S7 and S8 topologies, which are obtained only for positive dilatation rates, weaken with an increase in Karlovitz number and for the broken reaction zones regime flame the enstrophy transport is governed by S1, S2 and S4 topologies which are obtained for conditions irrespective of the value of dilatation rate. Thus, simple experimental configurations satisfying canonical features of S7, S8 and S1–S4 are likely to be necessary and sufficient in order to extract fundamental information regarding the enstrophy transport in the corrugated flamelets regime, whereas a simple configuration with predominant features of S1, S2 and S4 will be sufficient for physical understanding of the enstrophy transport in the broken reaction zones regime.

**Table 2:** Leading contributions of different flow topologies to the different terms of the mean enstrophy transport equation (eq. 5).

Case	${\overline{{\boldsymbol{T}}}}_{{\boldsymbol{I}}}$	${\overline{{\boldsymbol{T}}}}_{{\boldsymbol{I}}{\boldsymbol{I}}}$	${\overline{{\boldsymbol{T}}}}_{{\boldsymbol{I}}{\boldsymbol{I}}{\boldsymbol{I}}}$	${\overline{{\boldsymbol{T}}}}_{{\boldsymbol{I}}{\boldsymbol{V}}}$	${\overline{{\boldsymbol{T}}}}_{{\boldsymbol{V}}}$
A	S1–S4, S7	S2, S7, S8, S4	S4, S1, S2, S7, S3	S2, S7, S1, S4, S8	S2, S7, S1, S4, S8
B	S1, S2, S4	S1, S2, S8, S7, S4	S4, S1, S2	S7, S2, S1, S4, S8	S2, S7, S1, S1, S8
C	S1, S2, S4	S4, S1, S2	S4, S1, S2	S4, S1, S7	S4, S1, S7

## Summary

An existing three-dimensional DNS database^21, 35^ containing three freely propagating statistically planar *H*_2_-air flames representing typical flame-turbulence interaction in the corrugated flamelets, thin reaction zones and broken reaction zones regimes of turbulent premixed combustion has been used to analyse the enstrophy transport conditional on flow topologies within the flame. The flow topologies have been characterized in terms of three invariants of velocity gradient tensor (*P*, *Q* and *R*), where the first invariant is the negative of dilatation rate and second and third invariants can be linked to strain rate and enstrophy, and their generation mechanisms^2, 3, 11^. In this analysis the flow topologies have been categorized in 8 types (i.e. S1-S8) depending on the location of velocity gradient tensor in *P* − *Q* − *R* space^2, 3^. It has been found that the weakening of dilatation rate with increasing Karlovitz number plays a key role in the enstrophy transport in turbulent premixed flames. The contributions to the enstrophy transport conditional on topology have been analysed in detail and it has been found that the enstrophy generation due to baroclinic torque weakens from the corrugated flamelets regime case to the broken reaction zones regime case. Further, the flow topologies S1–S4, which can be obtained for all values of dilatation rate, contribute significantly to the enstrophy and its transport in the thin reaction zones and broken reaction zones regimes of premixed turbulent combustion. However, the topologies (i.e. S7 and S8), which are obtained only for positive values of dilatation rate, also contribute significantly to the enstrophy transport in the corrugated flamelets regime.

## Methodology

A three-dimensional complex chemistry (9 steps and 19 chemical reactions according to a detailed chemical mechanism^36^) compressible flow DNS database of freely-propagating statistically planar turbulent *H*_2_-air premixed flames with *ϕ *= 0.7 has been considered for this analysis. An equivalence ratio of 0.7 is chosen because an *H*_2_-air mixture for this equivalence ratio is known to be thermo-diffusively neutral^36^, such that the additional effects of the preferential diffusion are eliminated. Interested readers are referred to Im *et al*.^35^ and Wacks *et al*.^21^ for detailed information on the DNS database used for the current analysis and here a brief description of the numerical methodology is provided. The spatial discretisation is carried out using an 8^*th*^ order central difference scheme for internal grid points and the order of differentiation gradually decreases to a one-sided 4^*th*^ order scheme at the non-periodic boundaries. A fourth order Runge-Kutta scheme is used for explicit time advancement. The flame is initialised by a steady 1D planar laminar flame profile, and a pre-computed auxiliary divergence free, homogeneous, isotropic turbulence field generated using a pseudo-spectral method^37^ following Passot-Pouquet spectrum^38^ is injected through the inlet. The mean inlet velocity has been gradually modified to match turbulent flame speed as the simulation progresses. The temporal evolution of flame area has been monitored and the flame is taken to be statistically stationary when the flame area no longer varies with time. Turbulent inflow and outflow boundaries are specified in the direction of mean flame propagation and the transverse boundaries are taken to be periodic. The non-periodic boundaries are specified using an improved Navier Stokes characteristic boundary conditions (NSCBC) technique^39^.

The domain size is taken to be 20 mm × 10 mm × 10 mm (8 mm × 2 mm × 2 mm) in cases A and B (case C) and the domain has been discretised by a uniform Cartesian grid of 512 × 256 × 256 (1280 × 320 × 320). The grid spacing was determined by the flame resolution, ensuring about 10 grid points across *δ*_*th*_, and in all cases the Kolmogorov length scale remains bigger than the grid spacing (i.e. *η *≥ 1.5Δ*x* where *η* and Δ*x* are the Kolmogorov length scale and DNS grid spacing, respectively). Simulations have been carried out for 1.0*t*_*e*_, 6.8*t*_*e*_ and 6.7*t*_*e*_ (i.e. *t*_*e*_ = *l*_*T*_/*u* ^′^) for cases A-C respectively, and this simulation time remains comparable to several previous analyses^40–42^.

### Data availability

The datasets generated during and/or analysed during the current study are available from the corresponding author on reasonable request.

## References

[1] Tennekes, H. & Lumley, J. L. *A first course in turbulence* (MIT press, 1972).

[2] Perry AE, Chong MS (1987). A description of eddying motions and flow patterns using critical-point concepts. Annual Review of Fluid Mechanics.

[3] Chong MS, Perry AE, Cantwell BJ (1990). A general classification of three-dimensional flow fields. Physics of Fluids A: Fluid Dynamics.

[4] Soria J, Sondergaard R, Cantwell B, Chong M, Perry A (1994). A study of the fine-scale motions of incompressible time-developing mixing layers. Physics of Fluids.

[5] Blackburn HM, Mansour NN, Cantwell BJ (1996). Topology of fine-scale motions in turbulent channel flow. Journal of Fluid Mechanics.

[6] Chong M (1998). Turbulence structures of wall-bounded shear flows found using dns data. Journal of Fluid Mechanics.

[7] Chacin JM, Cantwell BJ (2000). Dynamics of a low reynolds number turbulent boundary layer. Journal of Fluid Mechanics.

[8] Ooi A, Martin J, Soria J, Chong M (1999). A study of the evolution and characteristics of the invariants of the velocity-gradient tensor in isotropic turbulence. Journal of Fluid Mechanics.

[9] Elsinga G, Marusic I (2010). Evolution and lifetimes of flow topology in a turbulent boundary layer. Physics of Fluids.

[10] Tsinober, A. Vortex stretching versus production of strain/dissipation. *Turbulence Structure and Vortex Dynamics* 164–191 (2000).

[11] Dopazo C, Martn J, Hierro J (2007). Local geometry of isoscalar surfaces. Physical Review E.

[12] Chen, J. H., Cantwell, B. J. & Mansour, N. N. The topology and vorticity dynamics of a three-dimensional plane compressible wake. In *Proc. Tenth Australasian Fluid Mechanics Conference*, 5–1 (1989).

[13] Sondergaard R, Chen J, Soria J, Cantwell B (1991). Local topology of small scale motions in turbulent shear flows. In 8th Symposium on Turbulent Shear Flows, Volume 1.

[14] Suman, S. & Girimaji, S. S. Velocity gradient invariants and local flow-field topology in compressible turbulence. *Journal of Turbulence* N2 (2010).

[15] Wang L, Lu X-Y (2012). Flow topology in compressible turbulent boundary layer. Journal of Fluid Mechanics.

[16] Tanahashi M, Fujimura M, Miyauchi T (2000). Coherent fine-scale eddies in turbulent premixed flames. Proceedings of the Combustion Institute.

[17] Chen, J. H., Yoo, C. S., Grout, R. & Gruber, A. Direct numerical simulation of flame stabilization downstream of a transverse fuel jet in cross-flow. Tech. Rep., Sandia National Laboratories (SNL-CA), Livermore, CA (United States) (2010).

[18] Cifuentes L, Dopazo C, Martin J, Jimenez C (2014). Local flow topologies and scalar structures in a turbulent premixed flame. Physics of Fluids.

[19] Cifuentes L, Dopazo C, Martin J, Domingo P, Vervisch L (2016). Effects of the local flow topologies upon the structure of a premixed methane-air turbulent jet flame. Flow, Turbulence and Combustion.

[20] Wacks D, Chakraborty N (2016). Flow topology and alignments of scalar gradients and vorticity in turbulent spray flames: A direct numerical simulation analysis. Fuel.

[21] Wacks DH, Chakraborty N, Klein M, Arias PG, Im HG (2016). Flow topologies in different regimes of premixed turbulent combustion: A direct numerical simulation analysis. Physical Review Fluids.

[22] Peters, N. *Turbulent combustion* (Cambridge university press, 2000).

[23] Hamlington PE, Poludnenko AY, Oran ES (2011). Interactions between turbulence and flames in premixed reacting flows. Physics of Fluids.

[24] Treurniet T, Nieuwstadt F, Boersma B (2006). Direct numerical simulation of homogeneous turbulence in combination with premixed combustion at low mach number modelled by the *G*-equation. Journal of Fluid Mechanics.

[25] Chakraborty N (2014). Statistics of vorticity alignment with local strain rates in turbulent premixed flame. European Journal of Mechanics B/Fluids.

[26] Lipatnikov A, Nishiki S, Hasegawa T (2014). A direct numerical simulation study of vorticity transformation in weakly turbulent premixed flames. Physics of Fluids.

[27] Chakraborty N, Konstantinou I, Lipatnikov A (2016). Effects of lewis number on vorticity and enstrophy transport in turbulent premixed flames. Physics of Fluids.

[28] Bobbitt B, Lapointe S, Blanquart G (2016). Vorticity transformation in high karlovitz number premixed flames. Physics of Fluids.

[29] Bobbitt B, Blanquart G (2016). Vorticity isotropy in high karlovitz number premixed flames. Physics of Fluids.

[30] Dopazo C, Cifuentes L, Chakraborty N (2017). Vorticity budgets in premixed combusting turbulent flows at different lewis numbers. Physics of Fluids.

[31] Moffatt H, Tsinober A (1992). Helicity in laminar and turbulent flow. Annual review of fluid mechanics.

[32] Steinberg AM, Driscoll JF, Ceccio SL (2008). Measurements of turbulent premixed flame dynamics using cinema stereoscopic piv. Experiments in Fluids.

[33] Steinberg AM, Driscoll JF (2009). Straining and wrinkling processes during turbulence–premixed flame interaction measured using temporally-resolved diagnostics. Combustion and Flame.

[34] Steinberg AM, Driscoll JF, Ceccio SL (2009). Three-dimensional temporally resolved measurements of turbulence–flame interactions using orthogonal-plane cinema-stereoscopic piv. Experiments in fluids.

[35] Im HG, Arias PG, Chaudhuri S, Uranakara HA (2016). Direct numerical simulations of statistically stationary turbulent premixed flames. Combustion Science and Technology.

[36] Burke MP, Chaos M, Ju Y, Dryer FL, Klippenstein SJ (2012). Comprehensive h2/o2 kinetic model for high-pressure combustion. International Journal of Chemical Kinetics.

[37] Rogallo, R. S. Numerical experiments in homogeneous turbulence. *NASA Technical Memorandum 81315* (1981).

[38] Passot T, Pouquet A (1987). Numerical simulation of compressible homogeneous flows in the turbulent regime. Journal of Fluid Mechanics.

[39] Yoo CS, Wang Y, Trouvé A, Im HG (2005). Characteristic boundary conditions for direct simulations of turbulent counterflow flames. Combustion Theory and Modelling.

[40] Han I, Huh KY (2008). Roles of displacement speed on evolution of flame surface density for different turbulent intensities and lewis numbers in turbulent premixed combustion. Combustion and Flame.

[41] Reddy H, Abraham J (2012). Two-dimensional direct numerical simulation evaluation of the flame-surface density model for flames developing from an ignition kernel in lean methane/air mixtures under engine conditions. Physics of Fluids.

[42] Dopazo C, Cifuentes L, Martin J, Jimenez C (2015). Strain rates normal to approaching iso-scalar surfaces in a turbulent premixed flame. Combustion and Flame.

